
JOURNAL INFORMATION
==============================
NLM Title Abbreviation: ScientificWorldJournal
Journal ID: ScientificWorldJournal
NLM Journal ID: 101131163
Journal ID: TSWJ

The Scientific World Journal

ISSN: 2356-6140
EISSN: 1537-744X
Publisher: Wiley

ARTICLE INFORMATION
==============================
PMCID: PMC6084173
PMID: 30147582
DOI: 10.1100/tsw.2001.131
Article ID: 656831
Article version: 1
Subjects: Short Report

Modulation of Apoptosis As a Therapeutic Strategy

Tomaselli Kevin J. *
Idun Pharmaceuticals, Inc., 11085 North Torrey Pines Rd., Suite 300, La Jolla, CA 92037, USA
*Kevin J. Tomaselli: ktomaselli@idun.com
Publication date: 2001
Electronic publication date: 2001 Oct 2
Volume: 1
First page: 104
Last page: 104
Copyright: Copyright © 2001 Kevin J. Tomaselli.
Copyright year: 2001
License: This is an open access article distributed under the Creative Commons Attribution License, which permits unrestricted use, distribution, and reproduction in any medium, provided the original work is properly cited.
License URL: https://creativecommons.org/licenses/by/3.0/

==============================
Miami *Nature Biotechnology* Short Reports
*TheScientificWorld* (2001) 1(S3), 104SR
ISSN 1532-2246; DOI 10.1100/TSW.2001.131

### MODULATION OF APOPTOSIS AS A THERAPEUTIC STRATEGY

Kevin J. Tomaselli, Ph.D.

Idun Pharmaceuticals, Inc., 11085 North Torrey Pines Rd., Suite 300, La Jolla, CA 92037;
(858) 623-1330; Fax (858) 625-2677
ktomaselli@idun.com

Cell death is a contributing factor in many human diseases. A significant proportion of the cell loss that occurs in tissue or organ injury and disease is by programmed cell death, or apoptosis. Apoptosis is a genetically controlled form of cell suicide effected by specific gene products acting within dying cells. A family of cysteine proteases, caspases, comprise the key effectors of the apoptotic pathway. Caspases are constitutively expressed in essentially all living cells as inactive zymogens. Following their interaction with caspase activator proteins (e.g. Apaf-1), caspases are converted to a catalytically active form, and cell death quickly ensues. Small molecule inhibitors of caspase proteases or caspase activator proteins represent a new approach to inhibiting cell death in the context of acute tissue and organ damage. The potential applications of apoptosis inhibitors for treating stroke, myocardial infarction and acute hepatic failure will be discussed.

### REFERENCES.

1. Mittl, P.R.E., Marco, S., Krebs, J., Bai, X., Karanewsky, D., Priestle, J., Tomaselli, K., and Grutter, M. (1997) J. Biol. Chem. 272, 6539-6547
2. Blanchard, H., Kodandapani, L., Mittl, P.R.E., Di Marco, S., Krebs, J.F., Wu, J.C., Tomaselli, K.J., and Grutter, M.G. (1999) Struct. Fold. Des. 7(9), 1125-1133
3. Hengartner, M.O. (2000) Nature 407, 770-776
